# Veterinary medical student perceptions of companion animal primary care as a career choice over an academic year

**DOI:** 10.3389/fvets.2022.989678

**Published:** 2022-09-16

**Authors:** Michael T. Nappier, Virginia K. Corrigan, Shelby Borowski, Danielle Lusk

**Affiliations:** ^1^Department of Small Animal Clinical Sciences, Virginia-Maryland College of Veterinary Medicine, Virginia Tech, Blacksburg, VA, United States; ^2^The Veterinary Technology Program, Department of Rural Resiliency and Innovation, Appalachian State University, Boone, NC, United States; ^3^Department of Population Health Sciences, Virginia-Maryland College of Veterinary Medicine, Virginia Tech, Blacksburg, VA, United States; ^4^The Edward Via College of Osteopathic Medicine, Blacksburg, VA, United States

**Keywords:** veterinary student, primary care, perception, career choice, general practice, companion animal

## Abstract

Despite companion animal primary care being the most common career choice for veterinarians, relatively little is known about students' perception of this career choice. In this study, the authors examined students' (233 at Time 1 and 119 at Time 2) perceptions of careers in companion animal primary care and whether perceptions changed over the course of an academic year or differed by year in veterinary school. The study was conducted by an online questionnaire sent to the student email listserv and the results analyzed by Mixed ANOVAs for each perception outcome. The study concluded that a majority of veterinary students have companion animal primary care as their preferred career choice and have a positive perception of it as a career choice. This positive perception increased over the course of an academic year, but did not differ significantly by year in school. First year students had a decrease in perception of level of training over time. This study sets a baseline for students' perceptions of companion animal primary care as a career choice at one college of veterinary medicine.

## Introduction

Primary care practitioners in any branch of medicine are the most numerous and frequent care providers. They stand on the proverbial front lines of practice and are usually the first sought after for consultation. This stands true in veterinary medicine where only approximately 10% of practicing veterinarians are identified as a board certified specialist by the American Veterinary Medical Association. Of the remaining 90% of veterinarians, approximately 75% of those practice in either companion animal exclusive or companion animal predominant primary care practices (1).

An interesting contrast is seen in many colleges of veterinary medicine. While a majority of graduates will go on to pursue a career in companion animal primary care, faculty teaching in colleges of veterinary medicine are often predominantly board-certified specialists and often have little to no experience in companion animal primary care. This is a result of the reliance on traditional veterinary teaching hospitals to provide the majority of the clinical experience for their students. The nature of these teaching hospitals has changed over the years, and they now provide mainly tertiary care specialty referral services. This gives students a significant exposure to difficult and complex case presentations and procedures. While this may be ideal for teaching specialty residents, it does not provide the necessary primary care/wellness caseload necessary for teaching new primary care veterinarians (2, 3). The structure of a veterinary teaching hospital is also oriented to facilitate referral cases so promotes a time management approach suited to those cases as opposed to primary care cases (2). Referral patients/clients have often booked appointments significantly in advance, may have traveled from a significant distance, and may be expecting to spend a significant portion of the day or even multiple days at the teaching hospital (3–6). This is not the case for primary care cases (7).

As Hubbell states, “the ideal case for educating veterinary students is far removed from the ideal case for educating specialists” (2).

The realization that colleges of veterinary medicine were graduating primarily new companion animal practitioners and yet giving them clinical experience that was more appropriate for specialty residents prompted a re-examination of veterinary clinical curricula (8). A renewed emphasis on primary care experience and competency based education has resulted at many colleges of veterinary medicine including the authors' (9). This idea of competency based education is not new but began to gain significant traction in the early 2000's particularly highlighted in the United States in the Association of American Veterinary Medical Colleges Foresight Project and the North American Veterinary Medical Education Consortium reports (10, 11). This emphasis on competency based education lead to a logical renewed emphasis on academic primary care as a means for developing competencies needed by the new graduates (12). To quote May, “Education of students in the culture and systems of specialist practice, either in the teaching hospital or private practice environment, is unlikely to be the best preparation for a career in primary health care, potentially creating dissonance as students move into their first jobs, with a loss of confidence in their ability and a lack of satisfaction in their work, even though, for many, this is the career that they have always sought” (12). The American Association of Veterinary Medical Colleges founded the Primary Care Veterinary Educators group formally in 2011 to connect and promote academic primary care educators.

These increased primary care experiences do not universally take place in a traditional teaching hospital primary care practice setting. Factors such as physical space and expense have led some colleges of veterinary medicine have primary care experiences in other locations than the teaching hospital. This may be primarily in a shelter medicine or distributed setting utilizing surrounding private practices to teach students (13, 14). These may also include service learning experiences such as Tufts at Tech or WisCARES (14, 15).

Despite companion animal primary care being the most common career choice for veterinarians, relatively little is known about students' perception of this career choice. Much more is known about students' career choices in underserved areas of veterinary medicine such as food/farm animal practice and practicing in rural areas (16, 17). Given the nature of the changing emphasis on companion animal primary care in colleges of veterinary medicine and the relative lack of understanding of students' perception of this career choice, the authors endeavored to examine students' perceptions of careers in companion animal primary care and whether perceptions changed over the course of an academic year or differed by year in veterinary school.

## Materials and methods

### Procedure and sample

**Figure 1:**
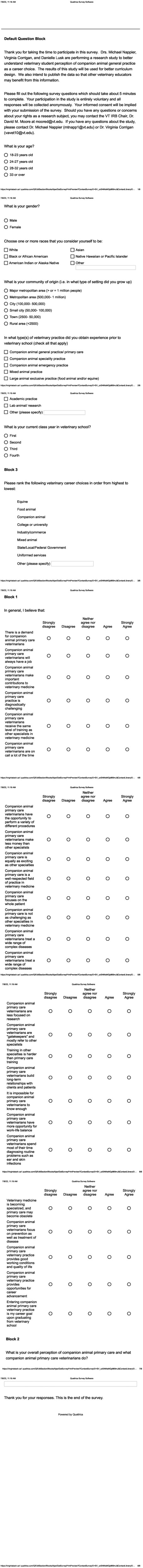
Veterinary student perceptions of primary care.

Veterinary students enrolled at Virginia Tech were recruited through a listserv to participate in this study. This study was carried out in accordance with the recommendations of the Virginia Tech Institutional Review Board, Protocol #17-136, with implied informed consent from all subjects in accordance with the Declaration of Helsinki. Data were collected online through a questionnaire at the beginning (referred to as Time 1 hereon) and end of the 2017-2018 academic year (referred to as Time 2 hereon) (Figure 1). Two-hundred and thirty-three veterinary students completed the questionnaire at Time 1, and 119 veterinary students completed the questionnaire at Time 2. Of these students, 74 students completed the questionnaire at both Time 1 and Time 2. Students were not compensated for their participation.

### Measures

#### Background information

Students were asked to report their age, gender, race, and community of origin (e.g., metropolitan, city, town, rural), and their year in veterinary school. To assess experience prior to veterinary school, students were asked, “In what type(s) of veterinary practice did you obtain experience prior to veterinary school (check all that apply)?” Students selected areas of experience from the following: Companion animal general practice/primary care, companion animal specialty practice, mixed animal practice, large animal exclusive practice (food animal and/or equine), academic practice, lab animal/research, and other. To assess student's preference for veterinary careers, students were asked to rank the following veterinary career choices from highest (1) to lowest (9): Equine, food animal, companion animal, college or university, industry/commerce, mixed animal, state/local/federal government, uniformed services, and other.

#### Perceptions of companion animal primary care

To measure perceptions of companion animal primary care, items from a previously developed measure of medical student attitudes toward family medicine (18) were revised for veterinary medicine. Additional questions were added by the research team. This measure assessed six aspects of primary care: positive perceptions, perceptions of work-life conditions, perceptions of training received relative to other specialists, perceptions of salary relative to other specialists, perceptions of focus on research, and level of interest in a companion animal primary care career.

#### Positive perceptions of companion animal primary care veterinarians

Using a 5-point Likert scale, 17 items assessed positive perceptions of companion animal primary care. Example items included, “In general, I believe that companion animal primary care veterinarians make important contributions to veterinary medicine” and “In general, I believe that companion animal primary care practice is diagnostically challenging.” Response options ranged from 1 = *strongly disagree* to 5 = *strongly agree*. Items were averaged, with possible scores ranging from 1 to 5. Higher scores represented more positive perceptions of companion animal primary care.

####  Perceptions of work-life conditions of companion animal primary care veterinarians

Using a 5-point Likert scale, three items were used to measure the perception of work-life conditions of companion animal primary care veterinarians. An example item was, “In general, I believe that companion animal primary care veterinarians have more opportunity for work-life balance.” Response options ranged from 1 = *strongly disagree* to 5 = *strongly agree*. Items were averaged, with possible scores ranging from 1 to 5. Higher scores represented more positive perceptions of work-life conditions of companion animal primary care.

#### Perceptions of training received relative to other specialists

To assess the perception of training received for primary care veterinarians relative to other specialists, students responded to the statement, “In general, I believe that companion animal primary care veterinarians receive the same level of training as other specialists in veterinary medicine.” Response options were 1 = *strongly disagree* to 5 = *strongly agree*. Higher scores indicated greater agreement that primary care veterinarians receive the same level of treatment compared to other specialists.

#### Perceptions of salary relative to other specialists

Using a 5-point Likert scale (response options 1 = *strongly disagree* to 5 = *strongly agree*), students responded to the statement, “In general, I believe that companion animal primary care veterinarians makes less money than other specialists.” Higher scores indicated greater agreement that primary care veterinarians make less money than other specialists.

#### Perceptions of focus on research

Students were asked to respond to the following item to assess perceptions of the focus on research for companion animal primary care veterinarians, “In general, I believe that companion animal primary care veterinarians are less focused on research,” with response options 1 = *strongly disagree* to 5 = *strongly agree*. Higher scores indicated greater agreement that primary care veterinarians are less focused on research.

#### Level of interest in companion primary care veterinary practice upon graduating

To assess the level of interest in companion primary care veterinary practice, students responded to the following item, “In general, I believe that entering companion animal primary care veterinary practice is my career goal upon graduating from veterinary school.” Response options were 1 = *strongly disagree* to 5 = *strongly agree*. Higher scores indicated higher level of interest in a companion animal primary care veterinary practice career.

### Data analysis

Data were analyzed in StataIC 16.0. All students with available data at each time point were included in the analyses. To check assumptions of normality for perception outcomes (i.e., sum score measures and single item measures), we examined histograms, skewness and kurtosis values, and also performed Shapiro-Walk tests. Results revealed that the data are approximately normal and thus were treated as continuously in all analyses. Descriptive statistics and bivariate correlations were examined to describe the sample and the associations between perceptions at each time point.

For perception measures, Mixed ANOVAs were analyzed for each perception outcome. Statistical significance was considered *p* ≤ 0.050. For significant effects, partial-eta squared values (η^2^) were reported to determine the size of the effect. The Mixed ANOVAs were used to examine two main effects (group membership; time) and one interaction effect (time x group membership). More specifically, the main effect of time specified if there was a significant change in overall mean scores for the entire sample from Time 1 to Time 2. This main effect does not consider group membership. The main effect of group membership (i.e., year in veterinary school) specified if overall mean scores between each of the four groups of students (1^st^, 2^nd^, 3^rd^, and 4^th^ year) were significantly different. This main effect does not consider time. Lastly, the Time x Group interaction considered both time and group membership. If the Time x Group interaction was statistically significant at the 0.05 level in the Mixed ANOVAs, simple effects were estimated through the Stata margins command. Specific simple effects examined were (1) if students significantly differed in their perception scores at each time point (i.e. Time 1, Time 2) and (2) if scores significantly increased/decreased for specific groups of students (i.e. 1^st^, 2^nd^, 3^rd^, and 4^th^ year) from Time 1 and Time 2. Main effects were not reported in the results if the interaction was significant.

## Results

### Demographic characteristics

At Time 1 (*N* = 233), the majority of students were female (*n* = 181, 77.7%), White (*n* = 204, 87.6%), and between 24–27 years old (*n* = 114, 48.9%) or 18–23 years old (*n* = 80, 34.3%). Fifty-seven (24.5%) students grew up in a metropolitan area (500,000 people or more), 33 (14.2%) grew up in a city (100,000 to 500,000 people), 45 (19.3%) grew up in a small city (50,000 to 100,000 people), 79 (33.9%) grew up in a town (2,500 to 50,000 people), and 19 (8.1%) grew up in a rural area (less than 2,500 people).

Similarly, at Time 2 (*N* = 119) the majority of students were female (*n* = 89, 74.8%), White (*n* = 104, 87.4%), and between 24–27 years old (*n* = 62, 52.1%) or 18–23 years old (*n* = 33, 27.7%). Thirty-one (26.1%) students grew up in a metropolitan area (500,000 people or more), 18 (15.1%) grew up in a city (100,000 to 500,000 people), 23 (19.3%) grew up in a small city (50,000 to 100,000 people), 34 (28.6%) grew up in a town (2,500 to 50,000 people), and 13 (10.9%) grew up in a rural area (less than 2,500 people).

### Veterinary-related characteristics

At Time 1, sixty-five (27.9%) students were in their first year of veterinary school, 62 (26.6%) were in their second year, 60 (25.8%) were in their third year, and 46 (19.7%) were in their fourth year. As for experience prior to veterinary school, the most commonly reported experience was companion animal general practice/primary care (*n* = 209, 89.7%), followed by lab animal/research (*n* = 82, 35.2%) and large animal exclusive practice (*n* = 79, 33.9%).

At Time 2, thirty-five (29.4%) students were in their first year of veterinary school, 19 (16.0%) were in their second year, 29 (24.4%) were in their third year, and 36 (30.2%) were in their fourth year. As for experience prior to veterinary school, the most commonly reported experience was companion animal general practice/primary care (*n* = 106, 89.1%), followed by large animal exclusive practice (*n* = 47, 39.5%) and lab animal/research (*n* = 43, 36.1%).

Veterinary career choice preferences at Time 1 and Time 2 for the students are displayed in Table 1. At Time 1 and Time 2, the most preferred careers (i.e., lowest scores) were companion animal, mixed animal, and college or university. The careers preferred the least (i.e. higher scores) at both time points were uniformed services and other (Table 1).

**Table 1:** Mean rank for veterinary careers at Time 1 and Time 2.

**Career**	**Time 1**	**Time 2**
	**Mean rank**	
Companion animal	2.49	2.30
Mixed animal	3.28	2.95
College or university	4.38	4.18
State/local/federal government	4.75	5.12
Food animal	5.00	5.13
Equine	5.27	5.03
Industry/commerce	5.40	5.48
Uniformed services	6.89	6.76
Other	7.54	8.04

Responses ranged from 1 to 9 (1 = *highest preference* and 9 = *lowest preference*).

### Perceptions of companion animal primary care

The association between the perception subscales for Time 1 and Time 2 for all students are displayed in Table 2. There were no significant differences in perceptions of primary care scores based on background characteristics. Mean scores for the four perception outcomes for first, second, third, and fourth year students, as well as the entire sample, are displayed in Tables 3, 4.

**Table 2:** Correlation matrix of perceptions of primary care measures at Time 1 and Time 2.

**Variables**	**1**	**2**	**3**	**4**	**5**	**6**	**7**	**8**	**9**	**10**	**11**	**12**
1. Positive T1	-											
2. Positive T2	0.67***	-										
3. Work-life T1	0.14*	0.25*	-									
4. Work-life T2	0.03	0.33***	0.61***	-								
5. Level of training T1	0.29***	0.18	0.06	−0.14	-							
6. Level of training T2	0.49***	0.38***	−0.01	0.02	0.40***	-						
7. Salary T1	−0.08	−0.06	−0.12	0.03	−0.33***	−0.10	-					
8. Salary T2	−0.32**	−0.05	−0.01	−0.11	−0.19	−0.32***	0.48***	-				
9. Research T1	−0.18**	−0.02	0.06	0.14	−0.18**	−0.24*	0.29***	0.12	-			
10. Research T2	−0.20	−0.24*	−0.08	−0.06	−0.07	−0.28**	0.15	0.03	0.43***	-		
11. Level of interest T1	0.33***	0.27*	0.16*	−0.02	0.02	0.08	0.14	0.13	−0.15*	0.08	-	
12. Level of interest T2	0.33**	0.42***	0.16	0.10	0.16	0.10	0.11	0.16	−0.11	−0.01	0.88***	-

^*^p ≤ 0.05,

^**^p ≤ 0.01,

^***^p ≤ 0.001.

**Table 3:** Average scores for positive, work-life conditions, and level of training perceptions by year in veterinary school.

	**Positive perceptions**			**Work-life conditions**			**Level of training**		
	**Time 1**	**Time 2**	**Overall mean**	**Time 1**	**Time 2**	**Overall mean**	**Time 1**	**Time 2**	**Overall mean**
First year students	3.79 (0.41)	3.82 (0.39)	3.81 (0.40)	3.61 (0.56)	3.59 (0.59)	3.60 (0.56)	3.43 (1.08)	2.70 (1.02)	3.07 (1.10)
Second year students	3.84 (0.48)	4.01 (0.44)	3.92 (0.46)	3.44 (0.98)	3.75 (0.75)	3.60 (0.88)	2.57 (1.08)	2.76 (1.04)	2.67 (1.05)
Third year students	3.73 (0.46)	3.90 (0.42)	3.82 (0.45)	3.21 (0.76)	3.28 (0.77)	3.25 (0.75)	2.84 (0.90)	2.84 (1.17)	2.84 (1.03)
Fourth year students	3.69 (0.36)	3.99 (0.16)	3.84 (0.32)	3.76 (0.60)	4.06 (0.70)	3.91 (0.65)	2.00 (0.89)	2.18 (1.08)	2.09 (0.97)
All Students	3.77 (0.43)	3.92 (0.39)	3.85 (0.42)	3.48 (0.76)	3.63 (0.73)	3.55 (0.75)	2.82 (1.10)	2.68 (1.07)	2.75 (1.09)

Responses ranged from 1 to 5 (1 = *strongly disagree* and 5 = *strongly agree*). Standard deviations are in parentheses. Overall mean refers to the average scores for each group across time.

**Table 4:** Average scores for salary, research, and level of interest perceptions by year in veterinary school.

	**Salary**			**Research**			**Level of interest**		
	**Time 1**	**Time 2**	**Overall Mean**	**Time 1**	**Time 2**	**Overall Mean**	**Time 1**	**Time 2**	**Overall Mean**
First year students	3.22 (0.80)	3.70 (1.02)	3.46 (0.94)	3.43 (0.90)	3.78 (0.67)	3.61 (0.80)	3.35 (1.15)	3.30 (1.26)	3.33 (1.19)
Second year students	3.62 (1.07)	3.71 (1.06)	3.67 (1.05)	3.43 (1.03)	3.38 (0.92)	3.40 (0.96)	3.10 (1.51)	3.43 (1.40)	3.26 (1.45)
Third year students	3.84 (0.69)	3.79 (0.98)	3.82 (0.83)	4.16 (0.69)	3.79 (0.71)	3.97 (0.72)	3.05 (1.54)	3.05 (1.51)	3.05 (1.51)
Fourth year students	3.73 (1.10)	3.82 (1.17)	3.77 (1.11)	4.09 (0.83)	4.18 (0.98)	4.14 (0.89)	2.64 (1.80)	3.00 (1.61)	2.82 (1.68)
All Students	3.57 (0.92)	3.74 (1.02)	3.66 (0.97)	3.72 (0.93)	3.73 (0.83)	3.72 (0.88)	3.09 (1.45)	3.23 (1.40)	3.16 (1.42)

Responses ranged from 1 to 5 (1 = *strongly disagree* and 5 = *strongly agree*). Standard deviations are in parentheses. Overall mean refers to the average scores for each group across time.

#### Positive perceptions

There was a significant main effect for time for positive perception scores. The average score across all participants at Time 1 was 3.75, which significantly increased to 3.90 at Time 2, *F*_(1, 57)_ = 13.02, *p* < 0.001. This was a large effect, η^2^ = 0.19. The main effect for year in veterinary school was not significant, *F*_(3, 57)_ = 1.12, *p* = 0.343. In other words, there was no significant differences in overall average positive perception scores between first, second, third, and fourth year students. When examining change over time by group membership, the Time x Group interaction was not significant, *F*_(3, 57)_ = 1.94, *p* = 0.133.

#### Work-life conditions

The main effect for time was not significant, *F*_(1, 57)_ = 3.68, *p* = 0.060. When examining overall group differences between year in veterinary school, the main effect was not significant, *F*_(3, 57)_ = 0.44, *p* = 0.723. Similarly, the Time x Group interaction was not significant, *F*_(3, 57)_ = 1.54, *p* = 0.215.

#### Level of training

The Time x Group interaction for perceptions of level of training was significant, *F*_(3, 57)_ = 3.72, *p* = 0.016, indicating differences from Time 1 to Time 2 by year in veterinary school for perceptions of training received compared to specialists. This was a medium sized effect, η^2^ = 0.16.

Simple effects showed differences among scores by group membership at Time 1, but not Time 2. More specifically, at Time 1, first year students had significantly higher levels of agreement that primary care veterinarians received the same level of training as specialists (*M* = 3.35) than third year students (*M* = 2.73) and fourth year students (*M* = 2.40), *t* = −3.43, *p* = 0.024 and *t* = −4.48, *p* = 0.001, respectively.

As for change over time, first year student's scores significantly decreased from Time 1 (*M* = 3.35) to Time 2 (*M* = 2.61), *t* = −2.96, *p* = 0.004. In other words, the level of agreement with the statement that primary care veterinarians receive the same level of training as specialists decreased for first year students over time. However, from Time 1 to Time 2, scores did not significantly increase or decrease for the second, third, or fourth year students.

#### Salary

The main effect for time was not significant, indicating no significant change in the overall salary perception score for all students from Time 1 to Time 2, *F*_(1, 57)_ = 0.15, *p* = 0.700. There was not a significant main effect for year in veterinary school, suggesting there were no differences between overall scores of first, second, third, and fourth year veterinary students in salary perception scores, *F*_(3, 57)_ = 1.58, *p* = 0.194. The Time x Group interaction was also not significant, *F*_(3, 57)_ = 1.90, *p* = 0.139.

#### Research

The main effect for time was not significant, *F*_(1, 57)_ = 0.12, *p* = 0.732, indicating no significant change in the average research perception score for all students from Time 1 to Time 2. There was not a significant main effect for year in veterinary school, suggesting no differences between overall scores of first, second, third, and fourth year veterinary students in research perception scores, *F*_(3, 57)_ = 0.52, *p* = 0.666. The Time x Group interaction was also not significant, *F*_(3, 57)_ = 2.16, *p* = 0.103.

#### Level of interest

There was a significant main effect for time for level of interest scores. The average score across all participants at Time 1 was 3.11, which significantly increased to 3.32 at Time 2, *F*_(1, 57)_ = 5.39, *p* = 0.024. This was a medium effect, η^2^ = 0.09. The main effect for year in veterinary school was not significant, *F*_(3, 57)_ = 0.04, *p* = 0.990, indicating no significant differences in scores by year in veterinary school. Similarly, the Time x Group interaction was not significant, *F*_(3, 57)_ = 1.93, *p* = 0.135.

## Discussion

The demographics of the study participants showed a clear majority were young, white, and female. This is unsurprising given the known demographics of veterinary school admissions (19). A strong majority of the participants also reported experience in companion animal general practice/primary care. This is also consistent with known demographics of practicing veterinarians as approximately 75% of all veterinarians in private practice categorize themselves as either companion animal exclusive or predominant (1).

At both time points, students most preferred career was companion animal primary care. Statistically significant differences were seen in questions of positive perceptions companion animal primary care over time. Students' answers revealed an overall positive increase in perceptions of companion animal primary care over the course of an academic year. The authors postulate that the increase in positive perceptions could be due to a larger opportunity for positive interactions with faculty in companion animal primary care during that portion of the curriculum (20, 21).

There was also significant change over time for first year students in their perceptions of training received. In the beginning of the academic year, first year students had the highest level of agreement that companion animal primary care veterinarians received the same level of training as other specialists. However, over time, their scores significantly decreased, indicating less agreement over the academic year. This may be due to increased exposure to specialty medicine in a tertiary care academic teaching hospital setting (22, 23).

No change in the perceptions of research in the primary care field was noted during the study period. However, a significant change in the level of interest in a career in companion animal primary care was seen. Over the course of the academic year students indicated that they became more interested in a career in companion animal primary care. Once again, the authors postulate that an increase in positive interactions with primary care faculty over the year (20).

When compared to other professional students in medical fields, veterinary students were somewhat more tempered in their perceptions toward primary care as a career option. In one study, students in a human medical program tended to rate the positive perceptions and work-life conditions of primary care higher and the negative perceptions and level of interest lower (18). Human medical students also tended to rate primary care as having a low prestige level, but high level of flexibility and positive work-life conditions (24–26). This is in contrast to human dental students who have an overall positive view of primary care/general dentistry with over 50% in one study electing to pursue general dentistry as a career as well as believing that general dentistry would “have the best future in terms of overall impact on the profession of dentistry” (27).

While this study contributes to the understanding of veterinary students' career choice perceptions, it does have several weaknesses. While it may have larger implications, this study is only directly applicable to this college of veterinary medicine. Each college of veterinary medicine has its own unique curriculum and composition of faculty members. The authors believe that these factors likely influence students' perception of career choices. More curricular time devoted to a specific field and/or outstanding faculty teaching or mentorship in a specific area would likely influence students' perceptions (28).

This study sets a baseline for students' perceptions of companion animal primary care as a career choice at one college of veterinary medicine. However, further research is needed to better characterize both individual students and other colleges of veterinary medicine. Possibilities for further investigation include repeating the study with at other colleges of veterinary medicine with different curriculum models to compare differences. A longitudinal cohort study following a single class of students through all 4 years would also provide additional data regarding changes in perception over time. A further interesting comparison would be looking at students' perceptions of primary care before and after graduation from veterinary school. Another avenue for investigation would be examining students' exposure to primary care faculty as mentors as well as in the curriculum.

This study shows veterinary students' perceptions of the positive and negative aspects as well as work-life conditions of companion animal primary as a career choice. Students' interest level in companion animal primary care remains tepid despite being the eventual career choice for a majority of practicing veterinarians. This reinforces the need for continued investment in companion animal primary care education and experiences as well as recruitment of strong primary care faculty and role models at colleges of veterinary medicine.

In conclusion, a majority of veterinary students have companion animal primary care as their preferred career choice and seem to have a positive perception of it as a career choice.

This positive perception increased over the course of an academic year, but did not differ significantly by year in school.

## Data availability statement

The raw data supporting the conclusions of this article will be made available by the authors, without undue reservation.

## Ethics statement

The studies involving human participants were reviewed and approved by Virginia Tech Institutional Review Board Protocol #17-136. The patients/participants provided their written informed consent to participate in this study.

## Author contributions

All authors substantial contributions to the conception or design of the work, or the acquisition, analysis, or interpretation of data for the work, drafting the work or revising it critically for important intellectual content, final approval of the version to be published, and agreement to be accountable for all aspects of the work in ensuring that questions related to the accuracy or integrity of any part of the work are appropriately investigated and resolved.

## Funding

Open access funding from Virginia Tech's University Library of $2000.

## Conflict of interest

The authors declare that the research was conducted in the absence of any commercial or financial relationships that could be construed as a potential conflict of interest.

## Publisher's note

All claims expressed in this article are solely those of the authors and do not necessarily represent those of their affiliated organizations, or those of the publisher, the editors and the reviewers. Any product that may be evaluated in this article, or claim that may be made by its manufacturer, is not guaranteed or endorsed by the publisher.

## References

[1] Market research statistics: U.S. veterinarians (2018). Available online at: https://www.avma.org/resources-tools/reports-statistics/market-research-statistics-us-veterinarians-2018 (accessed December 31, 2018).

[2] Hubbell JA. Veterinary teaching hospitals: current challenges and pathways for the future. J Vet Med Educ. (2008) 35:62–5. 10.3138/jvme.35.1.062 18339960

[3] Smith BP. Teaching the art of clinical practice: the veterinary medical teaching hospital, private practice, and other externships. J Vet Med Educ. (2003) 30:203–6. 10.3138/jvme.30.3.203 14658449

[4] Poon WYL Covington JP Dempsey LS Goetgeluck SL Marscher WF Morelli SC. Evaluation of a primary-care setting at a veterinary teaching hospital by a student business group: implementing business training within the curriculum. J Vet Med Educ. (2014) 41:189–96. 10.3138/jvme.0913-130R 24531532

[5] Weltman JG Prittie JE. The influence of a fast-track service on case flow and client satisfaction in a high-volume veterinary emergency department. J Vet Emerg Crit Care. (2021) 31:608–18. 10.1111/vec.13073 34297884

[6] Conner BJ Behar-Horenstein LS Su Y. Comparison of two clinical teaching models for veterinary emergency and critical care instruction. J Vet Med Educ. (2016) 43:58–63. 10.3138/jvme.0415-069R1 26751912

[7] Burge GD. Six barriers to veterinary career success. J Vet Med Educ. (2003) 30:1–4. 10.3138/jvme.30.1.1 12735308

[8] Eyre P. More on proposed changes to veterinary curriculum. Javma-J Am Vet Med A. (2011) 239:569. 10.2460/javma.239.5.569 21879953

[9] Coe JB. Primary care: an important role in the future of veterinary education. J Vet Med Educ. (2012) 39:209. 10.3138/jvme.0612-060 22940443

[10] Willis NG Monroe FA Potworowski JA Halbert G Vans BR Smith JF. Envisioning the future of veterinary medical education: The Association of American Veterinary Medical Colleges Foresight Project, final report. J Vet Med Educ. (2007) 34:3–42. 10.3138/jvme.34.1.120378881

[11] Schurig G Osburn BI. The north american veterinary medical education consortium (NAVMEC) looks to veterinary medical education for the future: “roadmap for veterinary medical education in the 21st century: responsive, collaborative, flexible” PREFACE. J Vet Med Educ. (2011) 38:320–6. 10.3138/jvme.38.4.320

[12] May S. Towards a scholarship of primary health care. Vet Rec. (2015) 176:677–82. 10.1136/vr.h2521 26113338

[13] Fuentealba C Mason RV Johnston SD. Community-based clinical veterinary education at Western University of Health Sciences. J Vet Med Educ. (2008) 35:34–42. 10.3138/jvme.35.1.034 18339954

[14] McCobb E Rozanski EA Malcolm EL Wolfus G Rush JE. A novel model for teaching primary care in a community practice setting: tufts at tech community veterinary clinic. J Vet Med Educ. (2018) 45:99–107. 10.3138/jvme.1116-174 28862534

[15] Alvarez E Lygo-Baker S Schultz K Gilles W Chun R. Veterinary and pharmacy students' expectations before and experiences after participating in an interdisciplinary access to care veterinary clinic, WisCARES. J Vet Med Educ. (2021) e20210006. 10.3138/jvme-2021-0006. [Epub ahead of print]. 34351831

[16] Elmore RG. Recruitment and retention of veterinary students for food animal practices. J Am Vet Med Assoc. (2003) 222:1697–9. 10.2460/javma.2003.222.1697 12830861

[17] Villarroel A McDonald SR Walker WL Kaiser L Dewell RD Dewell GA. Survey of reasons why veterinarians enter rural veterinary practice in the United States. Javma-J Am Vet Med A. (2010) 236:849–57. 10.2460/javma.236.8.849 20392180

[18] Zurro AM Villa JJ Hijar AM Tuduri XM Puime AO Alonso-Coello P. Medical student attitudes towards family medicine in Spain: a statewide analysis. BMC Fam Pract. (2012) 13:47. 10.1186/1471-2296-13-47 22642617 PMC3436784

[19] Mattson K. The Veterinary Student Population in Numbers. Javma-J Am Vet Med A. (2019) 254:1128. 10.2460/javma.254.10.1123

[20] Powers DE. Student perceptions of the first year of veterinary medical school. J Vet Med Educ. (2002) 29:227–30. 10.3138/jvme.29.4.227 12717641

[21] Cornish AR Caspar GL Collins T Degeling C Fawcett A Fisher AD. Career preferences and opinions on animal welfare and ethics: a survey of veterinary students in Australia and New Zealand. J Vet Med Educ. 43:310–20. 10.3138/jvme.0615-091R2 27153506

[22] Agathou S Stratis A Routh J Paramasivam SJ. Professional stereotypes among specialties and fields of work within the veterinary community. Vet Rec. (2022) e1486. 10.1002/vetr.1486. [Epub ahead of print]. 35257378

[23] Fish RE Griffith EH. Career attitudes of first-year veterinary students before and after a required course on veterinary careers. J Vet Med Educ. (2014) 41:243–52. 10.3138/jvme.0114-008R 24794169

[24] Selva Olid A Zurro AM Villa JJ Hijar AM Tuduri XM Puime AO. Medical students' perceptions and attitudes about family practice: a qualitative research synthesis. BMC Med Educ. (2012) 12:81. 10.1186/1472-6920-12-81 22909189 PMC3546071

[25] Creed PA Searle J Rogers ME. Medical specialty prestige and lifestyle preferences for medical students. Soc Sci Med. (2010) 71:1084–8. 10.1016/j.socscimed.2010.06.027 20674118

[26] Petchey R Williams J Baker M. 'Ending up a GP': a qualitative study of junior doctors' perceptions of general practice as a career. Fam Pract. (1997) 14:194–8. 10.1093/fampra/14.3.194 9201491

[27] Dhima M Petropoulos VC Han RK Kinnunen T Wright RF. Dental students' perceptions of dental specialties and factors influencing specialty and career choices. J Dent Educ. (2012) 76:562–73. 10.1002/j.0022-0337.2012.76.5.tb05290.x 22550102

[28] Roder CA May SA. The Hidden Curriculum of Veterinary Education: Mediators and Moderators of Its Effects. J Vet Med Educ. (2017) 44:542–51. 10.3138/jvme.0416-082 28876989

